# Intussusception around jejunal feeding tubes in pediatric patients: a retrospective two-center experience and management strategies

**DOI:** 10.1007/s00383-025-06209-1

**Published:** 2025-10-02

**Authors:** Nariman Mokhaberi, Omid Madadi-Sanjani, Lina Armbrust, Merle Körner, Johannes Görges, Daniel Tegtmeyer, Sebastian Schulz-Jürgensen, Konrad Reinshagen, Christian Tomuschat

**Affiliations:** 1 Department of Pediatric Surgery, University Medical Center Hamburg-Eppendorf, Hamburg, Germany; 2 Department of Pediatric Surgery, Hamburg Children’s Hospital Altona, Hamburg, Germany; 3 Department of Pediatric Radiology, Hamburg Children’s Hospital Altona, Hamburg, Germany; 4 Department of Pediatrics, University Medical Center Hamburg-Eppendorf, Hamburg, Germany

**Keywords:** Intussusception, Jejunal feeding, Feeding tube, Complication

## Abstract

**Introduction:**

Jejunal feeding via endoscopic, fluoroscopic, or surgical tube placement is widely used in pediatrics. A rare complication is intussusception around the jejunal feeding tube (JFT), which can cause obstruction or bowel ischemia. This study evaluated risk factors and management strategies.

**Methods:**

We retrospectively reviewed all children undergoing JFT placement or revision at two centers (2014–2025). Patient and procedural data were analyzed.

**Results:**

Among 58 patients (163 procedures), 7 children (12%) developed 8 episodes of intussusception. Median age at placement was 1 year (IQR 5); intussusception occurred a median of 1.16 years later (IQR 4.38). Five episodes resolved spontaneously, 2 by hydrostatic reduction, and 1 during unrelated surgery. Neurological impairment was present in 62% of patients. Kaplan–Meier analysis showed the highest risk within 1–2 years post-placement. Events occurred after endoscopic (6/43), surgical (1/13), and fluoroscopic (1/2) placements, with no clear link to technique.

**Conclusion:**

Intussusception around a JFT is rare but clinically significant. It should be suspected in children with abdominal pain, bilious vomiting, or feeding intolerance. Most cases can be managed non-surgically; our algorithm supports ultrasound diagnosis, observation or hydrostatic reduction, and surgery only for ischemia or failed conservative treatment.

## Introduction

Feeding via jejunal access is a well-established treatment in children in whom gastric feeding is not possible due to oral feeding intolerance, delayed emptying, or severe reflux. Jejunal feeding tubes (JFT) are frequently used in patients with neurological impairment and in patients with malignancies, dysphagia, or failure to thrive [1–3]. To bypass the problem of gastric and duodenal feeding, JFT should be placed approximately 40 cm distal to the Treitz ligament [4].

A recent position statement from the European Society for Paediatric Gastroenterology, Hepatology and Nutrition (ESPHGAN) recommends jejunal feeding in children with impaired oral or gastric feeding or with gastric outlet obstruction. Furthermore, it may be considered in some patients with severe gastroesophageal reflux disease (GERD) and risk of aspiration, especially in children with neurological impairment.

Depending on the expected duration of jejunal feeding, there are several options for placing JFTs. Nasojejunal tubes are an option in patients who are likely to receive short-term jejunal feeding (< 1 month). For longer periods, endoscopic or surgical JFT placement is indicated.

Endoscopic JFT may offer the option of simultaneous gastric access (percutaneous endoscopic gastrojejunostomy [PEG-J]/jejunostomy [PEJ]). Surgical JFT implantation can be performed via laparotomy or laparoscopy; however, it includes the opening of the intestinal lumen with potential contamination of the abdominal cavity. Most procedures include the fixation of the jejunum to the abdominal wall and oftentimes tunneling the catheter (Witzel’s technique or Roux-en-Y procedures, summarized here for brevity, are examples of surgical fixation methods). An alternative surgical approach is the Omega-Jejunostomy [5].

Postinterventional adverse events are mainly related to gastrointestinal, infectious, and mechanical complications. Mechanical complications include tube obstruction, dislodgement in the stomach, accidental tube removal, tube leakage, and bowel perforation or intussusception, a major complication with potentially significant morbidity and mortality [4–6].

In contrast to “classical” intussusceptions of the small intestine without an inserted feeding tube, which usually resolve spontaneously without intervention, intussusceptions around a feeding tube are unique circumstances that require an individual approach. However, the available data on pediatric patients with intussusceptions around JFTs comprise few retrospective studies and case reports, the majority of which deal with the genesis, diagnosis, and treatment of intussusception only briefly. There is still insufficient data on whether the type of intervention or other patient-specific factors are associated with an increased risk of intussusception. Moreover, the reported treatments differed from one another, and a treatment algorithm has not been suggested yet. Therefore, our study aimed to identify risk factors and suggest potential courses of management for intussusceptions around JFTs.

## Methods

### Data collection

This retrospective study included all patients with insertion or revision of a JFT between January 2014 and May 2025 at the Children’s Hospital of the University Medical Center Hamburg-Eppendorf (UKE) and the Altona Children’s Hospital Hamburg (AKK). Planned interval changes of JFTs were excluded.

Patients were identified through a retrospective chart review using the International Statistical Classification of Diseases and Related Health Problems (ICD) codes and the German procedure classification (OPS). Additionally, a re-examination of the radiological findings was conducted by two authors.

Patients were managed conservatively if clinically stable, without peritoneal signs, progressive symptoms, or laboratory evidence of ischemia (e.g., elevated lactate). Interventions were reserved for persistent intussusception or clinical deterioration.

### Statistics

Demographic and surgical data collected included age, sex, chronological data, underlying pathology, type of tube placement, symptoms at presentation, laboratory and radiological findings, and treatment of intussusception.

The primary endpoint was to determine the management of intestinal intussusception around the JFT. Secondary endpoints included identifying possible risk factors for developing an intussusception around a JFT and determining the time intervals between initial placement and intussusception, and between latest revision and intussusception.

Normally distributed variables are reported as mean ± standard deviation (SD), whereas non-normally distributed variables are reported as median with interquartile range (IQR).

Percentages are rounded to whole numbers.

This study adhered to the principles outlined in the Helsinki Declaration and received approval from the Hamburg Ethics Committee (2025–101440-BO-ff).

## Results

**Fig. 1:**
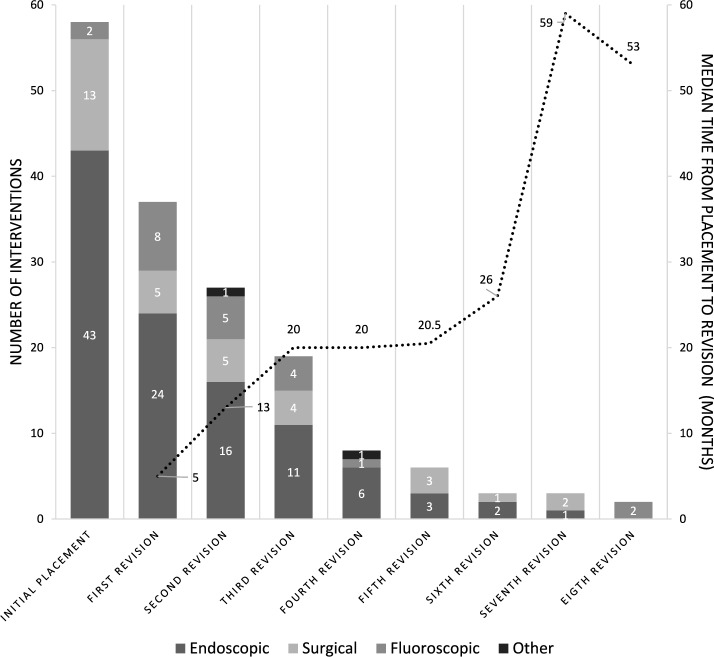
Number of interventions and median time to revision. Stacked bars show the number of interventions at each stage, categorized by approach (endoscopic, surgical, fluoroscopic, and others). The dotted line indicates the median time from initial placement to each revision

**Fig. 2:**
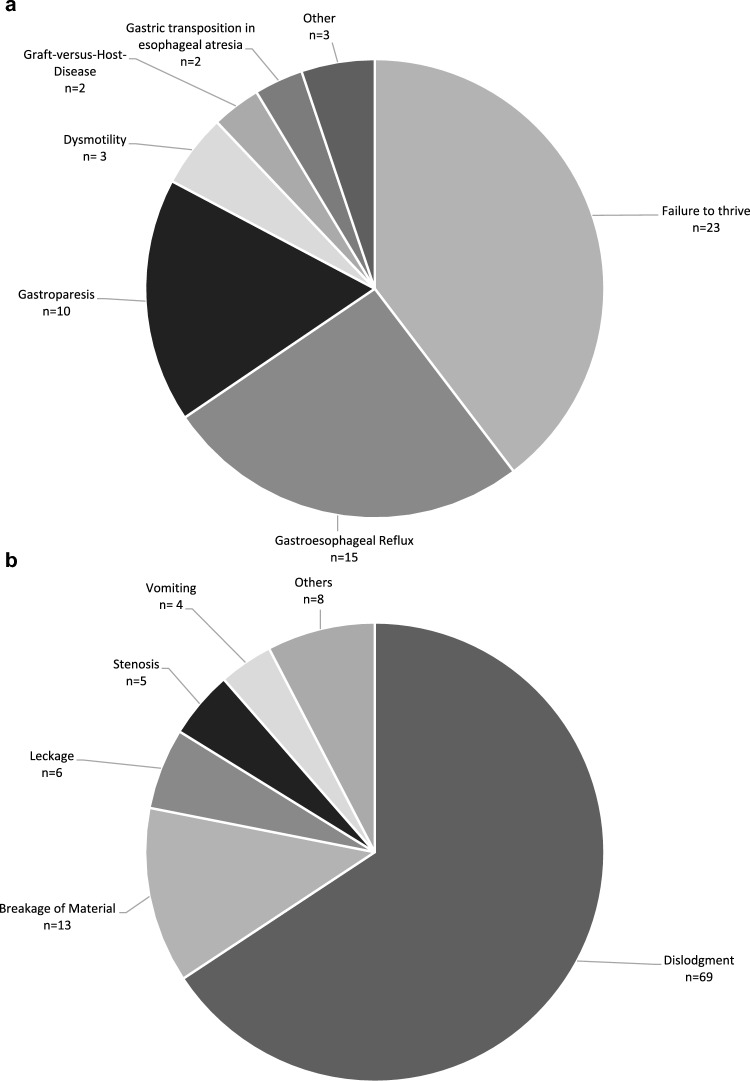
Total number of indications for an initial JFT placement and **b** subsequent JFT revisions

**Fig. 3:**
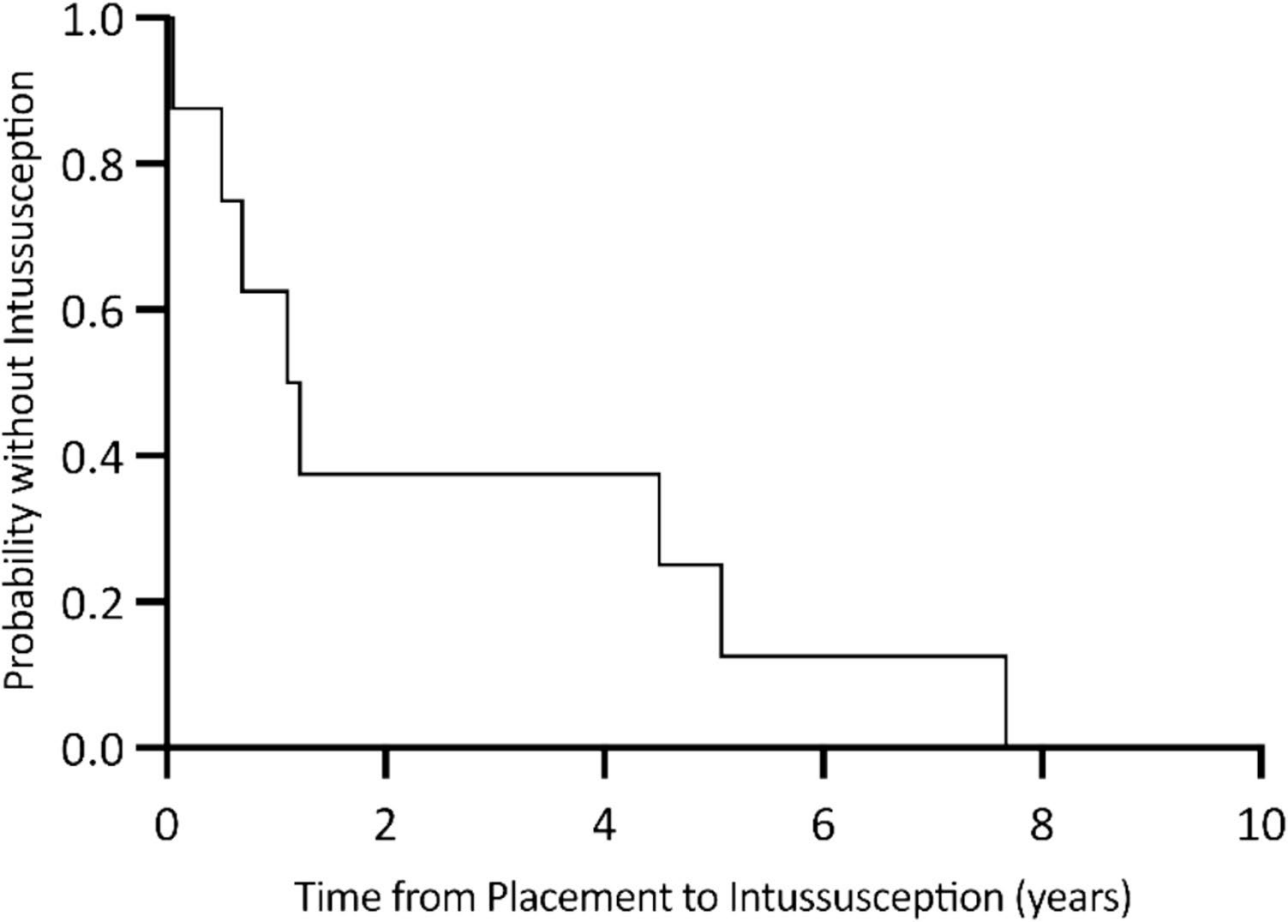
Kaplan–Meier Analysis: Time from Placement to Intussusception

163 JFT procedures were performed in 58 patients (33 male, 25 female) (Table 1; Figs. 1, 2 and 3). 43 (47%) were placed endoscopically (6 cases with intussusception), 13 (22%) surgically (1 case), and 2 (3%) fluoroscopically (1 case). Details and laboratory values can be seen in Table 2.

**Table 1:** Overview of JFT placements and revisions

*Sex (n)*	
Female	25
Male	33
*Total number of first placements (n)*	58
*Type of first placement (n) (%)*	
Endoscopic	43 (74)
Surgical	13 (22)
Fluoroscopic	2 (3)
*Age at first placement* *(years, median) (IQR)*	1 (5)
*Weight at first placement* *(kg, median) (IQR)*	9 (8.8)
*Total number of revisions (n)*	105
*Type of revisions (n) (%)*	
Endoscopic	63 (60)
Surgical	14 (13)
Fluoroscopic	26 (25)
Other*	2 (2)
*Number of patients with (n)*	
one revision	38
two revisions	26
three revisions	19
four revisions	9
five revisions	6
six revisions	3
seven revisions	3
eight revisions	2

^*^Seldinger Technique; manual repositioning

**Table 2:** Characteristics of the study’s patients with intussusceptions around JFTs

No.	Sex	Disease	Indication	Type of placement	Age at placement (years)	Age at intussusception (years)	Time from placement to intussusception (years)
1a	M	Dilated cardiomyopathy, VLCAD	Failure to thrive	endoscopic	1.61	2.11	0.50
1b^*^	M	“	“	“	1.61	2.71	1.10
2	F	H3F3B mutation	Failure to thrive, PEG-tube leakage	endoscopic	5.57	10.07	4.50
3	F	Progressive familial intrahepatic cholestasis (PFIC)	Feeding intolerance	surgical	0.92	8.58	7.67
4	M	T-acute lymphoblastic leukemia	Cachexia, Food refusal	radiologic	10.75	11.44	0.69
5	F	Bartter syndrome type III	Failure to thrive	endoscopic	0.53	5.59	5.07
6	M	Optic pathway glioma (OPG)	Failure to thrive, Food refusal	endoscopic	2.37	3.59	1.22
7	W	Unclear neuromuscular disease	Failure to thrive, Vomiting	endoscopic	0.93	0.99	0.06

No.	Sex	Symptoms	Diagnostics	Lactate^1^	CRP^2^	WBC^3^	Therapy
1a	M	Colicky abdominal pain for 3d	US	1.7 (c)	< 4	6.4	Spontaneous reduction
1b^*^	M	EHEC Infection	US	4.4 (v)	39	2.8	Manual reduction during open cholecystectomy for acute cholecystitis
2	F	Suspected bronchopneumonia, no abdominal symptoms	US	2.2 (v)	39	13.8	Spontaneous reduction
3	F	Diarrhea, Vomiting, Fever, Malaise	US	1.2 (c)	75	19.2	Hydrostatic reduction
4	M	Abdominal and back pain	US	1.2 (n/a)	< 2	9.0	Hydrostatic reduction
5	F	Increased vomiting after jejunal feeding, Diarrhea	US	1.1 (v)	< 4	22.3	Spontaneous reduction
6	M	Abdominal pain, increased vomiting, Diarrhea	US	0.9 (n/a)	64	7.5	Spontaneous reduction
7	W	Abdominal pain, Vomiting	US	1.2 (v)	< 4	13.7	Spontaneous reduction

^1^mmol/l + (c = capillary/ v = venous/ n/a = not available). ^2^c-reactive protein (mg/l). ^3^White blood cell count (cells/nl). ^*^Patient 1a and 1b are the same individual

### Demographic data

The median age at first placement was 1 year (IQR: 5), and median weight was 9 kg (IQR: 8.8). Neurological impairment was present in 62%.

### Intestinal intussusceptions around JFTs

The patient in whom two intussusceptions were detected during the study period underwent emergency surgery for acute cholecystitis during an intensive care stay one day after the second intussusception was detected. Concurrently, the intussusception was also reduced during the operation. The initial event of an intussusception reduced spontaneously. Most patients presented with abdominal pain, vomiting, or diarrhea. In all cases, the involved bowel segment could not be determined retrospectively from ultrasound images, and the distance of the tube tip from the ligament of Treitz was not documented. All cases were diagnosed via ultrasound. In the patients receiving conservative treatment, sonographic follow-up checks were performed at regular intervals of several hours. In five patients, spontaneous reduction was observed on the day of diagnosis.

In two patients, interventional reduction of the intussusception using hydrostasis was performed.

The patients with an intussusception underwent more JFT revisions compared to the JFT cohort (2.7 vs. 2.0). A Kaplan–Meier analysis demonstrated that most events occurred within 2 years post-placement (Fig. 3).

According to the recently validated Clavien–Madadi classification, the conservative management of intussusception around a JFT with hospitalization is classified as a Grade I event. The endoscopic and hydrostatic events performed under anesthesia are Grade IIIa events. It is difficult to define the surgical intervention as a Grade IIIb event according to Clavien–Madadi, as the procedure was performed for a concomitant disease not attributed to the intussusception [7, 8].

### Review of literature on intussusception around a JFT in pediatric patients

A review of the existing literature on pediatric patients with intussusceptions around a jejunal tube is presented in Table 3.

**Table 3:** Publications on pediatric patients with JFTs

Publicatio*n*	Patients (*n* =)	Placement	Intussusceptions (*n* =)	Symptoms	Diagnostics	Treatment	Follow-up
Kakiuchi et al. [13]	1	End.: 1	1	Urinary retention, convulsion, fever	CT	Surgery	n/a
McCann et al. [1]	197	NJ: 125/GJ: 51/Surg.:21 (REYJ)	4	n/a	n/a	3 conservative, 1 × Surgery	n/a
Singh et al. [22]	48	Rad.: 36/Surg.: 12 (Witzel: 7, REYJ: 4, MIS: 1)	1 (in Witzel Technique)	n/a	n/a	n/a	2.4 y (0.18–3.4) rad.; 1.8 y (0–3.5) surg
Morse et al. [6]	48	Rad.: 133/ Surg.: 8/End.: 4 Blind wire exchange: 13	2	Abdominal distension/crying and discomfort	US	n/a	1.1 y
Campwala et al. [20]	124	End.: 124	0	/	/	/	5.5 y (range: 1–9)
Picoraro et al. [9]	47	End.: 47	0	/	/	/	n/a
Egnell et al. [19]	33	Surg.: 33 (Witzel’s technique)	0	/	/	/	2.34 years (range: 0.27–12.6)
Jaskolska et al. [10]	38	Rad.: 38	1	n/a	n/a	Replacement of the JFT on the following day	n/a
Michaud et al. [23]	29	Rad.: 29	1	n/a	n/a	n/a	n/a
Fortunato et al. [2]	102	Rad.: 93/Surg.: 5/End.: 4	0	/	/	/	2 years (range: 0.1–18)
Hughes et al. [12]	251	Rad.: 251	40	Vomiting, abdominal pain, asymptomatic	US/Fluoroscopy with contrast	37 × Replacement, 2 × Air/Saline Reduction, 1 × Surgery	Not recorded
Connolly et al. [11]	5	Rad.: 5	7	Bilious vomiting, feeding intolerance	US/Fluoroscopy with contrast	4 × Change over wire, 2 × Air-/Contrast Reduction, 1 × Surgery	Not recorded

CT, Computed tomography; End., endoscopic; GJ, gastrojejunal; n/a, not available; MIS, minimally invasive surgery; NJ, nasojejunal; Rad., radiological; REYJ, Roux-en-Y-Jejunostomy; Surg., surgical; US, ultrasound

## Discussion

Intestinal intussusception around a jejunal feeding tube in pediatric patients is a rare but potentially dangerous complication due to the risk of developing mechanical ileus and intestinal ischemia. In contrast, mechanical complications, including dislocations, obstructions, and material breakage, are more prevalent issues in JFTs, which are inherently prone to complications. This factor also exerts a significant influence on the long-term outcome of the procedure [4]. To our knowledge, this is the largest retrospective two-center study reporting detailed diagnostic and therapeutic courses of JFT-associated intussusceptions in children.

A review of the existing literature reveals that cases of intussusception around JFTs have been documented to a lesser extent than in the present study (Table 3) [9, 10]. In accordance with the findings of this study, sonography has been demonstrated to be a reliable imaging diagnostic method [6, 11, 12]. Laboratory abnormalities were only mentioned in one of the studies referenced; this patient exhibited hyperreninemia, hyperaldosteronism, and hypokalemia because of urinary retention, as well as an increase in CRP levels over time [13].

Despite the available experiences in the literature, intestinal intussusceptions around JFTs were managed conservatively in the vast majority of our patients. In spite of the presence of abdominal symptoms and sonographic evidence of intussusception around the JFT, the absence of clinical and laboratory evidence of acute abdomen with intestinal ischemia permitted observation of the patients with close monitoring. No case of mechanical ileus with bowel ischemia, perforation, or necrosis was identified. The only surgical intervention compromised a simultaneous reduction of the intussusception in a case of concomitant acute cholecystitis and indication for cholecystectomy in a patient with prior endoscopic JFT insertion.

All other endoscopically placed JFTs showed spontaneous reduction without further interventions. The current literature describing cases of spontaneous reduction of intussusceptions around JFTs in pediatric patients does not specify the insertion techniques [1]. Therefore, this is the first report on successful conservative management of intussusceptions around JFTs in children with endoscopic insertion of a jejunal access.

In contrast, Kakiuchi et al. reported on a case of strangulated ileus requiring surgical reduction of an endoscopically inserted JFT [13]. In a systematic review, Morse et al. estimated the risk of intussusception per procedure to be 1.2%, excluding blind guidewire exchange, confirming a low but still significant risk of intussusception [6].

Our findings are also in contrast to the procedures described for adults, in whom surgical treatment of intussusceptions around JFTs is often necessary due to triggering pathologies, such as malignancies or chronic inflammatory bowel disease [14–18].

With regard to the diagnostic approach, in our cohort, ultrasound identified intussusception in all cases with no further need of additional imaging. However, recent reports included ultrasound, fluoroscopic contrast studies, and computer tomography [11, 13, 19]. The laboratory values exhibited no specific patterns; there were no consistent elevations in infection or lactate levels. Still, this observation is subject to the timing of the diagnosis and should be integral to the standard diagnostic process, nonetheless.

The majority of our cases presented with typical abdominal symptoms, such as pain, vomiting, and diarrhea. A limitation of our analysis is that it does not capture cases of intestinal intussusception in asymptomatic patients, which may have led to an underestimation of the true number of intussusceptions occurring around JTFs. However, reports from the literature on complications in patients with intussusceptions around JFTs mainly focus on symptomatic patients and their outcomes [6, 11, 13].

In our cohort, intussusception occurred a median of 1.16 years after initial JFT insertion. These results confirm recent reports that adverse events such as intestinal perforation can even occur a substantial time after tube insertion [20].

In our analysis, it however appears that the probability of intussusception increases significantly, particularly during the initial 1–2 years following initial JFT placement (Fig. 3).

As previously mentioned in the results, the cases in our cohort can be classified as minor complications according to the Clavien–Madadi classification, which incorporates non-medical errors and organizational problems into its classification of unexpected events in pediatric surgery [7, 8].

While in most institutions, the risk of intestinal intussusception around a JFT is often not recognized due to its rarity, there are various explanations and theories for the etiology. In a large cohort of 251 patients with radiologically placed JFTs, 40 intussusceptions in 30 patients were identified. The majority were treated by replacing the tube with either a standard or shortened JFT (*n* = 34) or a gastrostomy tube (*n* = 3); in two patients, an air/saline reduction was performed and the intussusception of the remaining patient was reduced surgically. Eight patients with a pigtail catheter had a recurrent intussusception, supporting the theory that the distal pigtail may catch the mucosa leading to intussusception. However, the authors also describe recurrent intussusception in pediatric patients with shortened and straight tubes. Thus, the type of tube is not considered to be the leading cause [12]. Also, in our heterogeneous cohort, no differences were observed since all of the JFTs were 9 French intestinal tubes.

Regarding the underlying diseases, it is speculated that muscle hypotonia as well as the size of the tube in relation to the bowel may be an additional risk factor [11]. In our cohort, one patient had a H3F3B mutation, which also results in reduced muscle tonus.

Another theory is that the feeding tubes cause chronic inflammation and the hypertrophied mucosa creates a lead point. This is conceivable but remains a theoretical approach without histological examination [21].

It is also important to consider that the surgical placement of the JFT usually involves the fixation of the jejunal loop to the abdominal wall. This action can serve as a pivot point for other intestinal loops, thereby promoting intussusception. This hypothesis is supported by the higher rate of volvulus observed in patients with surgically placed jejunal tubes. It was also demonstrated that major complications like an intussusception around a JFT were more frequently observed in REYJ. It is imperative that future research considers the surgical technique as a potential risk factor. Another possible risk factor for the development of intussusception is the number of revisions. Patients with intussusception underwent, on average, 2.7 revisions (no statistical testing was performed due to small sample size; data are presented descriptively) during the observation period, which is more than the average for the rest of our collective. Therefore, it can be posited that greater emphasis should be placed on the care and handling of the tube during insertion and maintenance to minimize unnecessary manipulation of the probe.

Another discussed theory (grouped under functional hypotheses) for the manifestation of the intussusception is the injecting force from the pump during feeding. It is postulated that it can drag the bowel, causing intussusception. As the majority of patients presented to our clinic in an emergency and due to the retrospective study design, this aspect could not be considered in our work. Nevertheless, this should be incorporated into prospective studies.

The available literature as well as our results illustrates the different courses of intussusceptions around JFT. As recommended by the ESPGHAN, there are different ways of inserting a jejunal tube. In our opinion, the approach should be tailored for each patient and primarily be done according to the expertise of the individual practitioner, with surgery generally being a more invasive process.

The study’s limitations can be attributed to its retrospective design and the small number of cases. Nevertheless, the present study represents one of the largest and most detailed collections of cases published to date, to our knowledge. In addition, the combination of the different types of placements by the various specialist departments (pediatric surgery, pediatric gastroenterology, adult gastroenterology, pediatric radiology) with the study design resulted in a highly varied documentation of the procedures, which led to a subsequent increase in the potential for error. Also, the potential for different treatment approaches cannot be excluded due to the interdisciplinary care provided; spontaneous reduction might also have occurred in the cases of interventional treatment for intussusceptions. Furthermore, asymptomatic transient intussusceptions could not be evaluated and may therefore have resulted in an underestimation of the true incidence.

## Conclusion and clinical algorithm

In conclusion, in our cohort, most JFT-associated intussusceptions in clinically stable children resolved without surgical intervention. Conservative observation with close sonographic monitoring was successful in the majority of cases. The routine diagnostic work-up of a patient with JFT and abdominal symptoms should always include an ultrasound and laboratory tests. If intussusception is detected, the decision depends on the patient's condition. In stable patients, a wait-and-see approach with regular checks is recommended. If spontaneous reduction does not occur or if the patient deteriorates, hydrostatic reduction, or endoscopic or fluoroscopic probe withdrawal can be attempted [14, 15]. Early intervention or surgical treatment should be avoided and only performed in signs of intestinal ischemia. Further prospective data are needed to establish optimal insertion techniques and risk prediction.

## Data Availability

No datasets were generated or analyzed during the current study.
